# Potential Therapeutic Improvements in Prostate Cancer Treatment Using Pencil Beam Scanning Proton Therapy with LET_d_ Optimization and Disease-Specific RBE Models

**DOI:** 10.3390/cancers16040780

**Published:** 2024-02-14

**Authors:** Michael Vieceli, Jiyeon Park, Wen Chien Hsi, Mo Saki, Nancy P. Mendenhall, Perry Johnson, Mark Artz

**Affiliations:** 1 University of Florida Health Proton Therapy Institute, Jacksonville, FL 32206, USA; michaelvieceli@ufl.edu (M.V.); jipark@floridaproton.org (J.P.); whsi@uams.edu (W.C.H.); msaki@floridaproton.org (M.S.); nmendenhall@floridaproton.org (N.P.M.); pbjohnson@floridaproton.org (P.J.); 2 Medical Physics Graduate Program, University of Florida College of Medicine, Gainesville, FL 32610, USA; 3 Department of Radiation Oncology, University of Florida College of Medicine, Gainesville, FL 32610, USA

**Keywords:** LET-optimized pencil beam scanning proton therapy, prostate cancer treatment, disease-specific/prostate-cancer-specific LET-dependent RBE modeling

## Abstract

**Simple Summary:**

LET-optimized pencil beam scanning (PBS) proton treatment plans have the potential to improve patient outcomes. Dose-escalated x-ray conformal therapy has improved the prostate cancer 5-year freedom from biochemical progression (FFBP) but has also increased gastrointestinal and genitourinary toxicities; toxicities can be reduced by escalating dose via a simultaneously-integrated boost (SIB) to the intraprostatic tumor (IPT). These techniques can be applied with greater precision using protons, which have shown high overall 5-year FFBP and low toxicities, although improvement in FFBP is needed for high-risk patients. Relative biological effectiveness (RBE) of 1.1 is uniformly assumed for proton treatment planning, although an increasing number of studies show that RBE increases with linear energy transfer (LET_d_). We show improvement with PBS LET-optimized plans over single-field optimized (SFO), or IPT-SIB plans through increased target volume LET_d_, RBE, and target-to-organs-at-risk (OAR) dose ratios (TODRs). This study also highlights the necessity of developing disease-specific LET-dependent RBE models.

**Abstract:**

Purpose: To demonstrate the feasibility of improving prostate cancer patient outcomes with PBS proton LET_d_ optimization. Methods: SFO, IPT-SIB, and LET-optimized plans were created for 12 patients, and generalized-tissue and disease-specific LET-dependent RBE models were applied. The mean LET_d_ in several structures was determined and used to calculate mean RBEs. LET_d_- and dose–volume histograms (LVHs/DVHs) are shown. TODRs were defined based on clinical dose goals and compared between plans. The impact of robust perturbations on LET_d_, TODRs, and DVH spread was evaluated. Results: LET_d_ optimization achieved statistically significant increased target volume LET_d_ of ~4 keV/µm compared to SFO and IPT-SIB LET_d_ of ~2 keV/µm while mitigating OAR LET_d_ increases. A disease-specific RBE model predicted target volume RBEs > 1.5 for LET-optimized plans, up to 18% higher than for SFO plans. LET-optimized target LVHs/DVHs showed a large increase not present in OARs. All RBE models showed a statistically significant increase in TODRs from SFO to IPT-SIB to LET-optimized plans. RBE = 1.1 does not accurately represent TODRs when using LET_d_ optimization. Robust evaluations demonstrated a trade-off between increased mean target LET_d_ and decreased DVH spread. Conclusion: The demonstration of improved TODRs provided via LET_d_ optimization shows potential for improved patient outcomes.

## 1. Introduction

Dose-escalation schemes are being increasingly used in order to achieve better tumor control in prostate cancer treatment [1,2,3]. For example, Dearnaley et al. [1] compared patient outcomes between groups treated with 2 Gy fractions of 64 Gy vs. 74 Gy in x-ray conformal therapy and found the 5-year freedom from biochemical progression (FFBP) to be 60% and 71%, respectively. However, they also found that late gastrointestinal (GI) and genitourinary (GU) grade 2+ toxicities were 33% and 11% in the dose-escalated group, respectively, compared to 24% and 8% in the lower-dose group. Thus, the challenge is to increase tumor control while minimizing organs-at-risk (OAR) toxicities. A response to this issue is to treat with a simultaneously integrated boost (SIB) to the intraprostatic tumor (IPT) [4,5]; the IPT is the site of common recurrence [6,7] and can be delineated using multiparametric MRI [8,9]. For a base dose of 77 Gy in 35 fractions with x-rays, Kerkmeijer et al. [4] found that the 5-year FFBP was 92% when the IPT was boosted to 95 Gy compared to 85% without the boost, while there were no statistically significant differences in grade 2+/3+ GI or GU toxicities. The most up-to-date review article that we found showed there is a general consensus that boosting the dose of the IPT increases tumor control without significant increases in OAR toxicities [10].

However, the nature of x-ray beams to deposit dose beyond the target results in irradiation of relatively large OAR volumes, placing a limit on the level of dose-escalation that can be given. In contrast, the increased dosimetric precision of proton therapy has been shown to achieve equivalent dose–volume histograms (DVHs) to the planning target volumes (PTVs) while lowering the OAR dose [11,12], increasing target-to-OAR dose ratios (TODRs) and expanding the potential for increased dose escalation. Several studies have investigated various treatment schemes for multiple patient outcome endpoints for proton therapy with escalated doses [13,14,15,16,17,18,19,20,21]. Zietman et al. [13] compared patient outcomes with a base dose of 50.4 Gy of x-rays with a double scattering proton boost of either 19.8 Gy(RBE) (assuming a relative biological effectiveness (RBE) of 1.1) or 28.8 Gy(RBE) and found the overall 5-year FFBP to be 78.8% and 91.3% in the conventional-dose and high-dose treatment arms, respectively, with no statistically significant increases in GU toxicities and only a small increase in grade 2 GI toxicities. A recent study by Bryant et al. [14] performed at our institution, the University of Florida Health Proton Therapy Institute (UFHPTI), prospectively investigated patient outcomes for 1327 prostate cancer patients who received between 78 Gy(RBE) and 82 Gy(RBE) to the PTV with single-field optimized (SFO) double scattering proton beams. The 5-year FFBP was 99% for low-risk patients, 94% for intermediate-risk patients, and 74% for high-risk patients. Meanwhile, only 5.4% of patients had grade 3+ GU toxicity, and 0.6% of patients had grade 3+ GI toxicity. However, the 5-year FFBP was only 55% for patients with a Gleason score of 9 and 42% for patients with T3-stage prostate cancer. In an effort to improve these results, another ongoing clinical study is being performed at UFHPTI, seeking to improve the therapeutic ratio by giving a SIB to the IPT.

The doses prescribed in proton therapy are specified as RBE-doses with a constant RBE of 1.1 [22,23]. However, there are an increasing number of studies that show that RBE increases with dose-weighted linear energy transfer (LET_d_) [24]. This provides much untapped potential for an RBE-dose boost based on the optimization of LET_d_ to yield the highest RBEs [25,26]. Studies have shown in example clinical proton treatment plans that LET_d_ distributions may be very different for the same dose distribution [27,28], and thus, there is the potential to optimize the LET_d_ while maintaining a similar physical dose or to optimize the LET-dependent RBE-dose [29,30,31,32,33]. The increased RBE-dose to the OARs must also be considered. LET_d_ is highest at the distal edge of the Bragg peak [24], with current clinical techniques having higher LET_d_ on average at the tumor periphery than within the tumor itself. As such, LET_d_ hotspots may exist in the OARs [27,28]. Although treatment plans using RBE = 1.1 may not include unacceptable hotspots in OARs if RBE is truly LET-dependent, current clinical plans may be delivering LET-based RBE-dose hotspots in OARs. LET_d_ optimization or LET-dependent RBE-dose optimization has the potential to both escalate the RBE-dose to target volumes and remove LET-dependent RBE-dose hotspots in OARs and so could increase TODRs and further improve patient outcomes beyond those obtainable with dose-optimization alone.

To demonstrate the potential for improved patient outcomes with LET_d_ optimization, we created LET-optimized, IPT-SIB, and SFO plans with a research build of RayStation 12B (RaySearch Laboratories, Stockholm, Sweden) for sample patients from the ongoing UFHPTI IPT-SIB clinical trial. To show the potential biological effects of plan LET_d_ on RBE and TODRs, we used several different LET-dependent RBE models ranging from generalized-tissue to disease-specific models. The LET_d_ between plan techniques was tested for statistical significance. LET_d_-volume histograms (LVHs) and DVHs are shown for an example patient to compare plan techniques and RBE models. The mean LET_d_ from all patients was used to calculate RBEs for each plan technique, and the RBE model was used to show the effects of LET_d_ on RBE. TODRs, defined based on clinical dose goals, were calculated and tested for statistically significant differences between plan techniques within the scope of each RBE model. Density uncertainty robustness was evaluated for an example patient as a function of LET_d_ and compared to TODRs and a minimum average RBE-dose (i.e., the average RBE-dose for an increased density perturbation).

## 2. Materials and Methods

### 2.1. Prostate Patients and Structure Delineations

Twelve patients with T1-T3b adenocarcinoma prostate cancer were selected from the UFHPTI IPT-SIB clinical outcome trial for this study; the sample size is consistent with other similar studies and provides sufficient statistical power to detect significant differences in dosimetric endpoints [34,35,36]. Radiation oncologists reviewed all patient workups, pathological grades, and prostate-specific antigen levels to verify the diagnosis. All patient data were anonymized and approved by an institutional review board (IRB). Sequential two-phase treatment plans were created for each patient to treat the prostate and seminal vesicles (PSV) with and without delivering pelvic nodal dose. The clinical target volume (CTV) was defined as the prostate, including extracapsular extension of the tumor and the proximal or entire seminal vesicles. The PTV was defined as the CTV plus 4 mm isotropic margin other than 6 mm expansion superiorly and inferiorly. An additional 1 mm margin was added to the pelvic nodal regions. The high-risk IPT was defined using 1.5 T to 3.0 T multiparametric MRI.

### 2.2. Treatment Plans and LET_d_ Optimization

Two lateral opposing proton beams were used for prostate pencil beam scanning (PBS) plans. SFO, IPT-SIB, and LET-optimized plans were created for each patient in the RayStation 12B treatment planning system, which includes the ability to specify LET_d_ optimization objectives. All plans prescribed 78 Gy(RBE) in 39 fractions (using RBE = 1.1) to the PTV PSV and 46 Gy(RBE) in 23 fractions to the pelvic nodes. The IPT simultaneously received a boost dose of 85.8 Gy(RBE) in 39 fractions in IPT-SIB plans. The CTV PSV and PTV nodes were robustly optimized using a 0.2 cm isotropic uncertainty in the patient setup position and 2% density uncertainty [37]. For LET-optimized plans, the LET_d_ objectives used were min LET_d_ of 4.5 keV/μm to the CTV PSV, max LET_d_ of 3.2 keV/μm to the bladder with a dose threshold of 22 Gy(RBE), and max LET_d_ of 2.5 keV/μm to the rectum with a dose threshold of 20 Gy(RBE). The RayStation Monte Carlo dose engine was used to calculate the dose and LET_d_ with 50,000 ions/spot for plan optimization and 0.5% uncertainty for the final dose calculation; the accuracy of the dose engine has been benchmarked against FLUKA [38]. 

### 2.3. RBE Models

We used two LET-dependent RBE models that were used in a previous study from our institution in the application of breast cancer [34]. The first model is a linear LET-weighted RBE model from McMahon et al. [39]. The McMahon RBE is calculated as

(1) $${\mathrm{RBE}}_{McM}=1+\kappa {\mathrm{LET}}_{d}$$

where κ = 0.055 µm/keV is a constant fit to cell survival data from two cell lines [40,41] and is consistent with the optimal κ-factor that minimized the variability in the McNamara modeled RBE-dose (described below) in example treatment plans for α/βs ranging from 2 Gy to 10 Gy, and so Equation (1) can be considered a generalized-tissue model. The second LET-dependent model used is a phenomenological RBE model from McNamara et al. [42]. The McNamara model uses 287 data points of x-ray and proton α/βs with respect to LET_d_ for a wide variety of animal and human cells, which Paganetti et al. [24] collected in a comprehensive literature review. The RBE is calculated as

(2) $${\mathrm{RBE}}_{McN}=\frac{1}{2D_{p}}\left(\sqrt{{\left(\frac{\alpha }{\beta }\right)}_{x}^{2}+4D_{p}{\left(\frac{\alpha }{\beta }\right)}_{x}RBE_{max}+4D_{p}^{2}RBE_{min}^{2}}-{\left(\frac{\alpha }{\beta }\right)}_{x}\right)$$

where $D_{p}$ is the physical proton dose per fraction, ${\left(\alpha /\beta \right)}_{x}$ is for x-rays, and ${RBE}_{max}$ and ${RBE}_{min}$ are the asymptotic values of RBE as $D_{p}\to 0$ and $D_{p}\to \infty$ [43], respectively, and are calculated as

(3) $${RBE}_{max}=0.99064+\frac{0.35605}{{\left(\alpha /\beta \right)}_{x}}{\mathrm{LET}}_{d}\;{RBE}_{min}=1.1012-0.0038703\sqrt{{\left(\alpha /\beta \right)}_{x}}{\mathrm{LET}}_{d}.$$

This shows that ${\mathrm{RBE}}_{McN}$ increases with LET_d_, decreases with ${\left(\alpha /\beta \right)}_{x}$, and decreases with dose per fraction. When the computed RBE in a voxel was greater than ${RBE}_{max}$ (Equation (3)) (e.g., $D_{p}$ in the voxel is really small), the ${\mathrm{RBE}}_{McN}$ was set equal to ${RBE}_{max}$, and when the computed RBE was less than ${RBE}_{min}$, ${\mathrm{RBE}}_{McN}$ was set equal to $RBE_{min}$. The ${\left(\alpha /\beta \right)}_{x}$s we used in this study were 1.5 Gy for the prostate/PTV PSV [44], 5.4 Gy for the rectum [44,45], and 5 Gy for the bladder [44].

**Figure 1:**
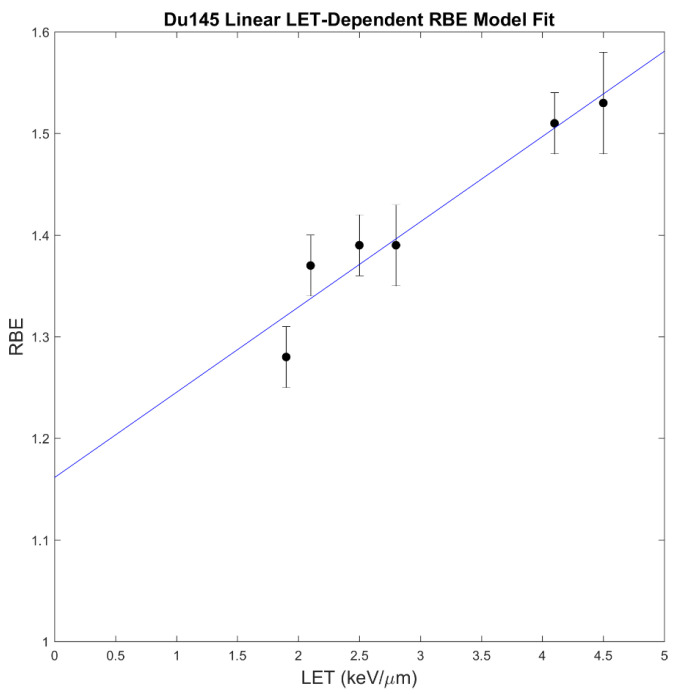
Du145 (human prostate cancer) linear LET-dependent RBE model fit ($R^{2}$ = 0.93). Data source: Mara et al. [46].

However, many of the data points used to fit the McNamara model are from animal cells, and only two data points are from human prostate cancer (Du145) at one LET_d_ value [24] (the McMahon model is not informed by prostate cancer data). Multiple studies have shown that the McNamara model underestimates the RBE in Du145 prostate cancer cells [46,47]. To make an estimate of prostate-cancer-specific RBE, we fit a linear model to Du145 cell survival data from Mara et al. [46], using the values of expRBE_10_ (experimental RBE for 10% survival) from Table II (Mara et al. [46]). The Du145 model was determined to be

(4) $${\mathrm{RBE}}_{Du145}=1.16+0.084{\mathrm{LET}}_{d}$$

with a coefficient of determination of $R^{2}$ = 0.93, and the model fit is shown below in Figure 1. The Du145 model was only applied to target volumes and not OARs since it is prostate-cancer-specific.

### 2.4. Statistical Analysis and Evaluation

Statistical tests of significance were performed on the differences in LET_d_ between plan techniques. LET_d_ was evaluated in the PTV PSV, IPT, prostate, and volume receiving 70 Gy(RBE = 1.1) or more (V70) regions of the bladder and rectum since volumes receiving higher doses have been shown to be predictive of toxicity [21]. A bootstrap method [48] of 10,000 iterations was used to randomly sample with replacement from the 12 patients (1) the mean voxel-wise differences in LET_d_ for the target volumes and (2) the differences in mean LET_d_ to the V70 regions for the OAR structures to empirically estimate the distributions for the metrics. From the bootstrap samples, the means were calculated, and the 95% confidence intervals (CI) were empirically determined as the interval spanning the 2.5th to 97.5th percentiles. Because 15 comparisons were made, the Bonferroni–Holm correction for multiple comparisons was applied to control the family-wise error rate [49], and the CIs were redetermined at the Bonferroni–Holm-corrected confidence levels (family-wise confidence level of 84.5%). The results were statistically significant if 0 was not in the CI [50].

The mean LET_d_ in the aforementioned structures for each patient was bootstrapped across all patients with 10,000 iterations to obtain the mean and 95% CI of the mean LET_d_. These were plugged into the three RBE models to calculate the mean RBE and 95% CI for each plan; for the McNamara model, which depends on the dose per fraction, the prescription dose was assumed (and since the V70 regions are adjacent to the target volume, an assumed average dose between 70 Gy(RBE) and 78 Gy(RBE) results in 1.9 Gy(RBE) per fraction).

The LVHs and DVHs for an example patient were exported from RayStation, with the PTV PSV, IPT, prostate, bladder, and rectum structures evaluated. DVHs comparing treatment plan techniques are shown for the McMahon model, plus the Du145 model for the target volumes. DVHs comparing the LET-dependent RBE models to RBE = 1.1 for the LET-optimized plan are also shown.

The therapeutic ratio is quantified as the tumor control probability (TCP) divided by normal tissue complication probability (NTCP). However, a study by Pederson et al. showed that NTCP models fit with x-ray data inaccurately predicted proton rectal toxicity [51]. As an alternate metric that considers clinically relevant dose goals and RBE, as well as being intuitive and straightforward to use in treatment planning, we defined the target-to-OAR dose ratio (TODR) as

(5) $${\mathrm{TODR}}_{bladder}=\frac{D95_{PTV\;PSV}}{D20_{bladder}}$$

for the bladder and

(6) $${\mathrm{TODR}}_{rectum}=\frac{D95_{PTV\;PSV}}{D10_{rectum}}$$

for the rectum where DX represents the dose that X% of the structure volume is at least receiving, and DX is taken from DVHs. Equations (5) and (6) represent how much dose can be given to the D95 region of the PTV PSV per unit dose to bladder D20 or rectum D10 regions, respectively. These definitions of TODR were defined based on the following clinical dose goals: (1) at least 95% of the PTV PSV should receive 100% of the prescription dose; (2) no more than 20% of the bladder should receive 70 Gy or higher; and (3) no more than 10% of the rectum should receive 70 Gy or higher. When calculating the TODRs with the Du145 model, since it is prostate-cancer-specific and not applicable to the OARs, the McNamara bladder D20s and rectum D10s were used.

The pairwise differences in TODR for each patient, separately for both the bladder and rectum, were tested for statistical significance. A bootstrap method of 10,000 iterations was used to get the mean pairwise difference in TODR and 95% CI for all patients. Comparisons were made between plan techniques within each of the four RBE models, including RBE = 1.1. With 12 comparisons each, the Bonferroni–Holm correction was applied separately for the bladder and rectum TODRs, resulting in a family-wise confidence level of 85.46% each.

Additionally, the TODRs were bootstrapped (10,000 iterations) over all 12 patients to calculate the mean TODRs and 95% CI for each plan technique within the scope of each RBE model. As an evaluation of the RBE models, the percent differences in TODR between RBE models for a given plan were calculated.

### 2.5. Robust Evaluation

To investigate the effects of density uncertainty on LET_d_ optimization, we performed robust evaluations for an example patient; a more detailed analysis on robustness and LET_d_ optimization for carbon ion beams is presented by Fredriksson et al. [52]. Fredriksson et al. describe an LET_d_ trilemma in which improving two of the factors of range robustness, uniform dose, and high LET_d_ comes at the expense of the third factor. This is due to LET-optimized plans creating the target dose using the gradient of the distal edges of the individual beams. Because the gradient of the distal edge is linear, density perturbations will result in the dose to the whole field being increased or decreased uniformly. The treatment plans in this present study emphasize uniform dose and high LET_d_, which results in larger DVH deviations from the nominal DVH with density perturbations than for SFO plans. As such, we investigated how density uncertainty robustness can be improved by allowing the LET_d_ to decrease by implementing robust min/max DVH constraints in optimization. Only the phase without pelvic nodal irradiation was evaluated, with the prescribed dose being 32 Gy(RBE = 1.1) for this phase (for this reason, it is not appropriate to directly compare TODRs or RBE-doses to the other data presented in this study, which consider both phases, except that plans described earlier were re-evaluated here for only the non-nodal phase). Using the same robust optimization conditions of 2% density uncertainty and 0.2 cm isotropic setup uncertainty, a robust min DVH constraint of 32 Gy(RBE) to 95% volume of the CTV PSV, max DVH of 32 Gy(RBE) to 95% volume, min DVH of 33 Gy(RBE) to 50% volume, and max DVH of 33 Gy(RBE) to 50% volume were used. With this being considered 0% tolerance, the tolerance of the min/max DVH constraints from the nominal 32/33 Gy(RBE) was increased in several increments (e.g., 5% would have constraints of min/max DVH of 30.40/33.60 Gy(RBE) to 95% volume and 31.35/34.65 Gy(RBE) to 50% volume); loosening the tolerance indirectly allows the LET_d_ to increase. Dose/LET_d_ density perturbations of ±2% were calculated; setup uncertainty perturbations were not included since their effect was much less significant than density perturbations. DVH spread was calculated as the D95 dose (RBE = 1.1) of the +2% density DVH minus that of the −2% DVH divided by twice that of the nominal DVH. Du145 TODRs were calculated according to Equations (5) and (6) with the McNamara model used for the OARs. The minimum average RBE-dose to the CTV PSV, which occurs with the +2% density perturbation, was calculated for each LET-dependent RBE model according to Equations (1)–(4) using the average dose and average LET_d_ in the +2% density perturbation scenario. D95 DVH spread, TODR_bladder_ and TODR_rectum_, and minimum average RBE-dose to the CTV PSV were all evaluated as a function of the nominal LET_d_. A four-beam technique was also considered to see if robustness with high LET_d_ could be improved, using both the same objectives as the original LET-optimized plan and with the robust min/max DVH constraints, with gantry angles of 50°, 130°, 230°, and 310°.

## 3. Results

**Figure 2:**
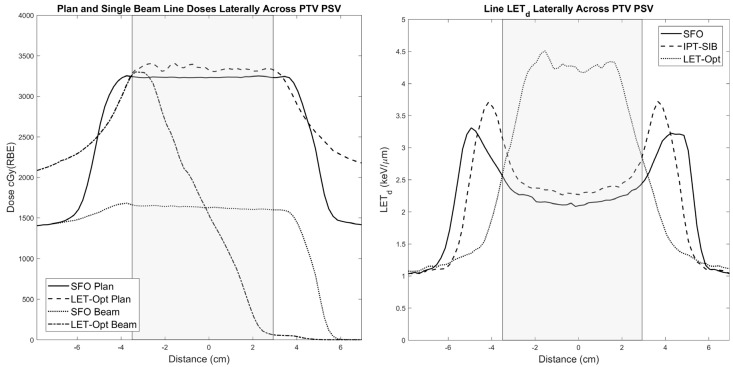
(**Left**) Single-beam and total plan line doses for SFO and LET-optimized plans. (**Right**) Total line LET_d_ for all three plans. The PTV PSV is represented by the shaded gray region. These line profiles correspond to the CT plan images in Figure 3.

**Figure 3:**
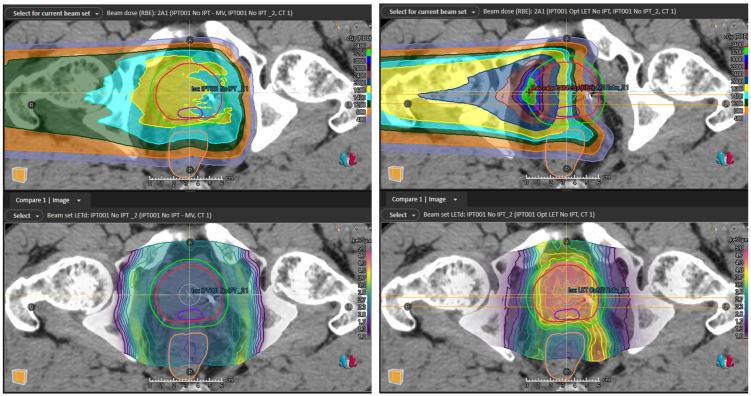
(**Top left**) Single beam from SFO plan. The beams shoot through the PTV and place high LET_d_ outside the target (**bottom left**, shown for both parallel opposed beams). (**Top right**) Single beam from LET-optimized plan. The beams abut in the center of the CTV PSV to achieve high LET_d_ and shoot through the rectum to minimize its LET_d_ (**bottom right**, shown for both parallel opposed beams). The IPT is delineated by the purple contour, the prostate/CTV PSV by the red contour, the PTV PSV by the green contour, and the rectum by the brown contour.

SFO, IPT-SIB, and LET-optimized plans were created for 12 patients; the prescribed dose (RBE = 1.1) was met for all plans, and bladder and rectum V70 (RBE = 1.1) regions were below the clinical goals of 20% and 10% volume, respectively, for all plans. The SFO beam has a uniform modulation across the PTV PSV, which, when combined with the opposing beam, results in a highly uniform field (Figure 2 left). Doing this places the distal edge behind the PTV, placing higher LET_d_ outside the PTV and lower LET_d_ inside the PTV (Figure 2 right and Figure 3 bottom left). The IPT-SIB plan is similar, with the distal edge LET_d_ peaks being drawn inward closer to the PTV and the LET_d_ being slightly higher overall due to a higher weighting of the Bragg peaks used to boost the dose to the IPT (Figure 2 right). The LET-optimized beam, however, abuts its distal edge in the center of the PTV (Figure 2 left and Figure 3 upper right), with its gradient dropping off across the PTV (Figure 2 left). When combined with the opposing beam, it produces a flat field across the PTV with less but acceptable uniformity than the SFO plan (Figure 2 left). The advantage of the LET-optimized technique is that it creates a field of high LET_d_ inside the PTV, and the LET_d_ drops outside the PTV (Figure 2 right and Figure 3 bottom right), in contrast to the SFO and IPT-SIB plans. Furthermore, the LET-optimized beam shoots through the rectum (Figure 3 upper right), which keeps the LET_d_ in the rectum low and places the high LET_d_ beyond the rectum (Figure 3 bottom right).

**Figure 4:**
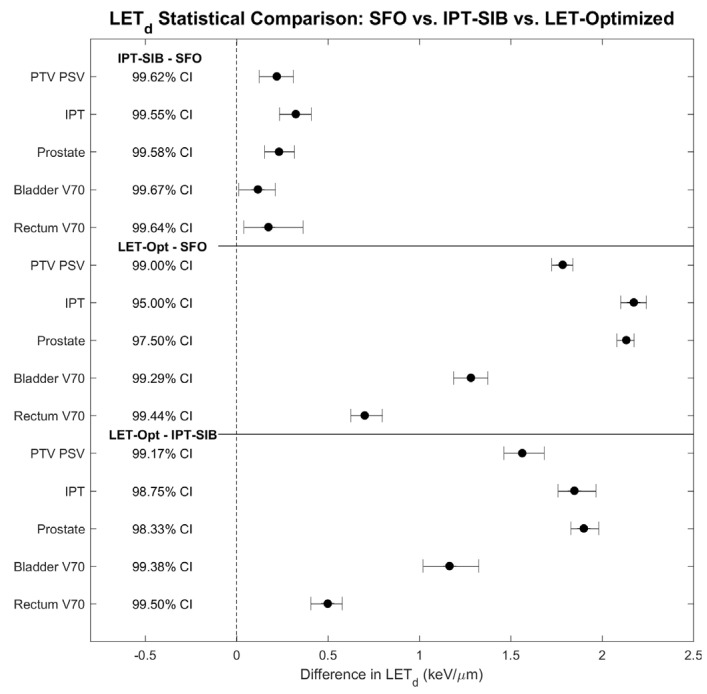
Statistical tests of significance for difference in LET_d_ between plans. All comparisons showed statistical significance at the Bonferroni–Holm-corrected confidence levels (shown in the figure). A comparison was deemed statistically significant if the confidence interval did not cross 0. The family-wise confidence level is 84.5%. CI: confidence interval.

All comparisons of LET_d_ between plan techniques showed statistically significant differences at the Bonferroni–Holm family-wise confidence level of 84.5% (Figure 4).

The mean LET_d_ and RBEs with 95% CIs are shown below in Table 1. The mean LET_d_ increased from ~2.2 keV/µm in the SFO plans to ~2.4 keV/µm in the IPT-SIB plans to ~4.2 keV/µm in the LET-optimized plans. While the IPT-SIB plan may result in about a 2–3% increase in target volume RBE over the SFO plan, the LET-optimized plan may result in increases in RBE from 11% up to 18% over the SFO plan with RBEs above 1.5 in the Du145 model. Assuming the prescription dose of 78 Gy(RBE = 1.1), the Du145 model predicts that the IPT-SIB plan would only result in a mean increase of 1.39 Gy(RBE) over the SFO plan in the prostate (due to RBE, not considering the physical dose boost), while the LET-optimized plan would result in 12.71 Gy(RBE) over the SFO plan, which is clinically significant. Note that these increases in RBE-dose are not relative to the RBE = 1.1 prescription (i.e., they are not increases over 78 Gy(RBE)) and must be interpreted in the context of the RBE model absolute dose predictions. The OAR RBEs remained near 1.1, with the greatest difference between target volume and OAR RBEs occurring in the LET-optimized plans. While LET_d_ increased in the OAR V70 regions, the size of the V70 regions generally decreased by single-digit percentages for the rectum.

**Figure 5:**
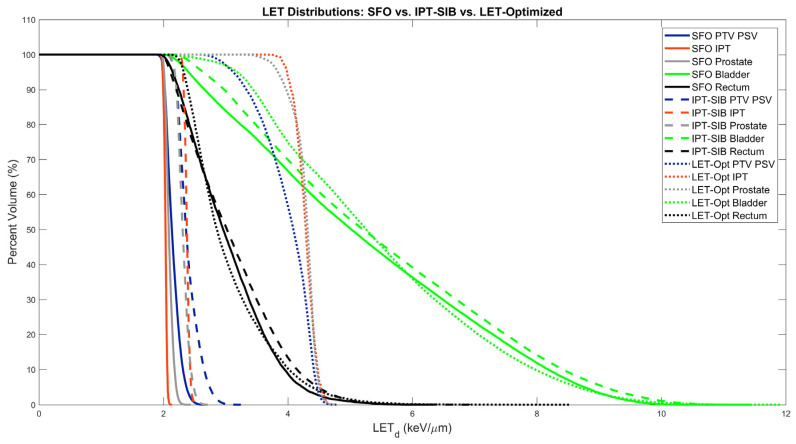
LET_d_-volume histograms (LVHs) for an example patient.

The LVHs for all three plans for the example patient shown in Figure 2 and Figure 3 are shown below in Figure 5. While an increase in the target volume of LVHs is evident for the IPT-SIB plan over the SFO plan, the increase is prominent for the LET-optimized plan. The bladder LVHs, and to a lesser extent, the rectum LVHs, extend to much higher LET_d_ than the target volumes; however, inspection of the treatment plans reveals that the high LET_d_ is in regions of low to negligible dose. While LET_d_ in the OAR V70 regions were statistically significant across all patients, the LVHs in the example patient show that OAR LET_d_ is similar overall other than at higher bladder volumes. Overall, Figure 5 shows that the LET_d_ to target volumes can greatly be increased without having nearly as large an effect on OARs.

**Figure 6:**
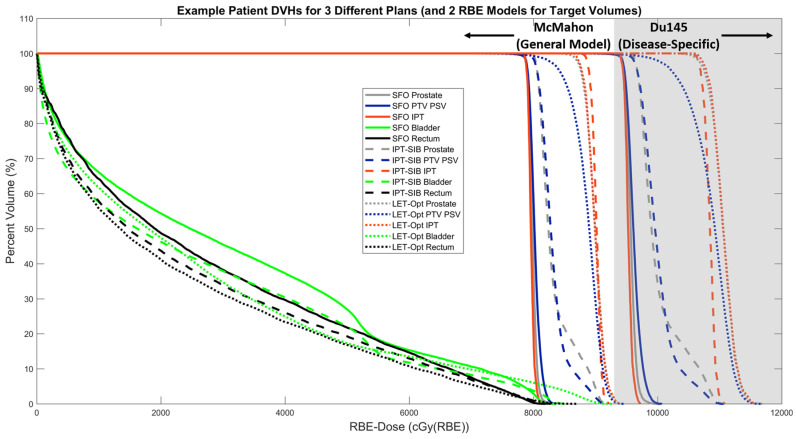
Dose–volume histograms (DVHs) for SFO, IPT-SIB, and LET-optimized plans using the McMahon model plus the Du145 model for target volumes for an example patient. The gray-shaded region at higher doses is used to visually differentiate between the Du145 DVHs and the McMahon DVHs, although all DVHs are on the same scale.

Figure 6 shows the DVHs for all three treatment plans for the example patient using the McMahon model plus the Du145 model for target volumes. For both RBE models, the IPT-SIB plan results in DVH increases in the target volumes over the SFO plan, and the LET-optimized plan results in a much greater increase over both. The absolute increases in DVHs are greater in the Du145 model than in the McMahon model, demonstrating the importance of considering the RBE model when comparing plans. The increase in the IPT DVH in the IPT-SIB plan compared to the prostate and PTV PSV is due to the physical dose boost to the IPT, making the IPT DVH comparable to the LET-optimized target volume DVHs. The different plans result in modest differences in the OAR DVHs, with the IPT-SIB and LET-optimized plans having lower DVHs than the SFO plan. However, there are some low-volume hotspots in the bladder, which is likely due to a small volume of it being located in the PTV PSV. Overall, these DVHs show an improvement in the TODRs of the IPT-SIB plan over the SFO plan and an improvement over both plans for the LET-optimized plan.

**Figure 7:**
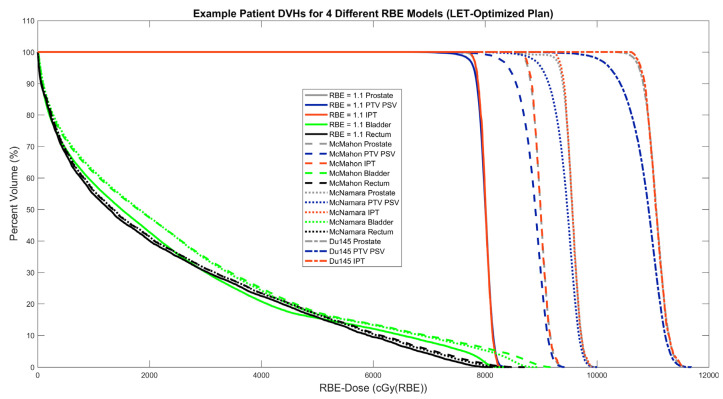
Dose–volume histograms (DVHs) for three LET-dependent RBE models (McMahon, McNamara, Du145) compared to RBE = 1.1 for an example patient LET-optimized plan.

Figure 7 shows DVHs for all three LET-dependent RBE models in reference to dose with RBE = 1.1 for the example patient LET-optimized plan. The most striking feature is the large increase in target volume doses predicted over the planned dose with RBE = 1.1, with the different models predicting an increase of about 10 Gy(RBE) to 30 Gy(RBE) in RBE-dose. Equally as conspicuous is the variability in predicted increase in the RBE-dose in the target volumes between models, with the McNamara model making higher predictions than the McMahon model and the Du145 model by far resulting in the highest RBE-dose. Meanwhile, the choice in the RBE model had little effect on the bladder and rectum DVHs, except the LET-dependent models predict higher hotspots in the bladder, which, as mentioned before, is partly in the PTV PSV.

**Figure 8:**
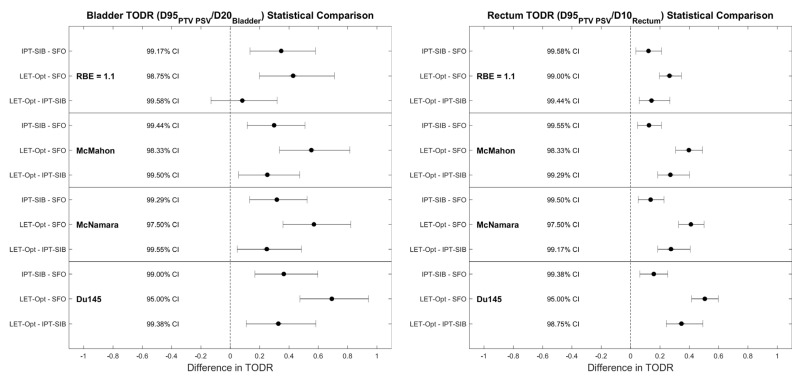
Statistical tests of significance for difference in (**left**) bladder TODR and (**right**) rectum TODR between plan techniques for each RBE model. All comparisons showed statistical significance at the Bonferroni–Holm-corrected confidence levels (shown in the figure), except for RBE = 1.1 IPT-SIB vs. LET-optimized for the bladder, which does not consider LET_d_. A comparison was deemed statistically significant if the confidence interval did not cross 0. The family-wise confidence level is 85.46%. CI: confidence interval.

Bladder and rectum TODRs were calculated for each patient, for each plan technique and RBE model, and tested for statistical significance (Figure 8). All except one of the bladder TODR comparisons showed a statistically significant difference at the family-wise confidence level of 85.46%; the only comparison that was not statistically significant was LET-optimized vs. IPT-SIB for RBE = 1.1, which does not consider LET_d_. All comparisons of the rectum TODR showed statistically significant differences.

**Figure 9:**
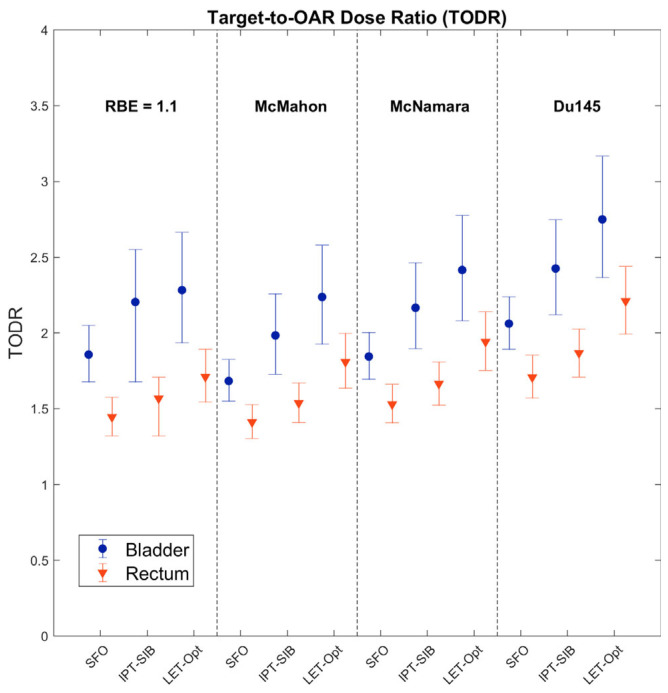
Bladder and rectum target-to-OAR dose ratios (TODRs). Note that the overlap of the confidence intervals is due to these comparisons being non-pairwise, while the statistically significant differences were determined with pairwise comparisons.

The TODRs are shown below in Figure 9. Although the magnitude of the TODRs varies between RBE models, the TODRs increase from SFO to IPT-SIB to LET-optimized plans regardless of which RBE model is used. Of note, the TODRs increased with plan technique for RBE = 1.1 (except IPT-SIB vs. LET-optimized for the bladder), demonstrating that some of the differences are due to the physical dose, which is expected for the IPT-SIB plan with the physical dose boost compared to the SFO plan; the physical dose to the rectum was generally reduced in the LET-optimized plans. For the bladder TODR, the percent difference between SFO and IPT-SIB plans is ~18% for all RBE models, with little difference between RBE = 1.1 and all three LET-dependent RBE models. However, the percent difference between SFO and LET-optimized plans is 23% for RBE = 1.1 and ~33% for LET-dependent models, and the percent difference between IPT-SIB and LET-optimized plans is 3.6% for RBE = 1.1 and ~12% for LET-dependent models. Similarly, for the rectum TODR, the percent difference was ~9% for both RBE = 1.1 and LET-dependent models for SFO vs. IPT-SIB. The percent difference between SFO and LET-optimized plans is 18.4% for RBE = 1.1, and ~28% for LET-dependent RBE models, and the percent difference between IPT-SIB and LET-optimized plans is 9.1% for RBE = 1.1 and ~18% for LET- dependent models. The variability in percent differences between LET-dependent RBE models was within 2.5% for each comparison. Thus, while RBE = 1.1 may approximate the percent difference in TODRs between SFO and IPT-SIB plans and so may potentially be able to correctly predict the percent difference in patient outcomes, RBE = 1.1 is insufficient when considering LET_d_ optimization.

**Figure 10:**
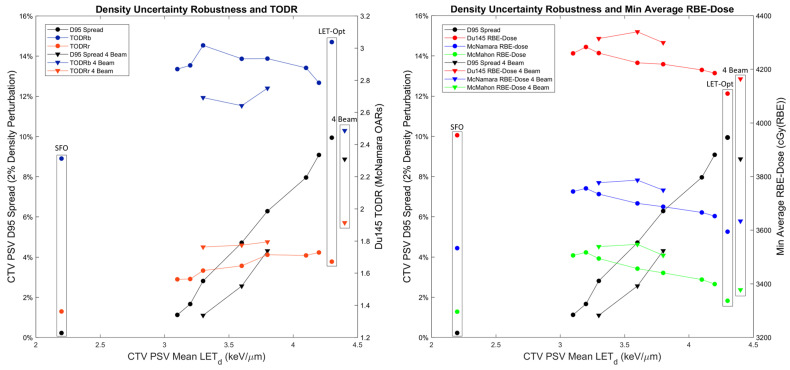
(**Left**) CTV PSV D95 spread (left y-axis) and Du145 TODRs (right y-axis) as a function of CTV PSV nominal mean LET_d_. The Du145 TODRs were calculated with the McNamara OAR RBE-doses since Du145 is prostate-cancer-specific. (**Right**) CTV PSV D95 spread (left y-axis) and minimum average RBE-dose (right y-axis) evaluated for all three LET-dependent RBE models as a function of CTV PSV nominal mean LET_d_. Labeled with the encompassing boxes, these quantities in both plots were evaluated as single data points for the SFO plan, the LET-optimized plan with the original objectives used throughout the rest of this study, and the four-beam LET-optimized plan using said original objectives.

Density uncertainty robustness as quantified by the D95 DVH spread, varied by adjusting robust min/max DVH constraints to the CTV PSV, is evaluated as a function of the resulting LET_d_ and compared to the TODRs (Figure 10 left) and minimum average RBE-dose (i.e., the low RBE-dose scenario that occurs with the +2% density perturbation) for the three LET-dependent RBE models (Figure 10 right) for an example patient. The D95 spread decreases with LET_d_, meaning that robustness improves with decreased LET_d_. The rectum TODR also decreases with decreasing LET_d_, likewise meaning that the rectum TODR decreases with improved robustness (there is no distinct pattern for the bladder TODR for this example patient). However, all the evaluated points, while being less robust than SFO plans, have increased TODRs over SFO plans. The minimum average RBE-dose increases with improved robustness/decreasing LET_d_. The highest two-beam Du145 minimum average RBE-dose occurred at 3.2 keV/μm and 4283 cGy(RBE) with an RBE = 1.1 D95 DVH spread of 1.66%. What is most significant, however, is that even as the minimum average RBE-dose decreases as higher LET_d_ is achieved, in all cases, it remains higher than that of SFO plans (which, due to its high level of robustness, is essentially equivalent to its nominal dose) for all LET-dependent RBE models. This means that even though the RBE = 1.1 dose may go below an acceptable deviation from prescription, the LET-dependent RBE-dose compensates for it, even when considering the adjusted LET-dependent RBE reference dose (which we define as the LET-dependent RBE-dose achieved via the SFO plan when its RBE = 1.1 prescription dose is met). This presents yet another reason why RBE = 1.1 is insufficient when considering LET_d_ optimization.

For the same objectives/constraints, the four-beam LET-optimized plans achieved higher LET_d_ (~0.1–0.3 keV/μm increase, which results in ~0.8–2.5% increased Du145 RBE). Both the rectum TODR and the minimum average RBE-dose increased over that of the two-beam plans. Significantly, these increases come with a reduced D95 spread. The bladder TODR decreases with four beams due to the angles at which the anterior oblique beams cross the bladder, but it is worth noting that (1) the TODRs considering nodal irradiation are already lower than the ones presented here for the non-nodal phase only for the same reason, in which case this effect from four beams may have a smaller impact, and (2) the beam angles could be further modified, or arc therapy may achieve optimal weightings for each beam angle, to increase the bladder TODR.

## 4. Discussion

### 4.1. Dosimetric Improvements and Clinical Potential

The amount of dose-escalation that can be given is constrained by the TODRs to limit toxicities to the OARs. Studies have shown that the 5-year FFBP has increased and grade 2+/3+ GI or GU toxicities have decreased moving from x-ray conformal plans [1,2,3] to x-ray IPT-SIB plans [4,5,10] to proton SFO plans [13,14], and further improvement is expected in the ongoing UFHPTI proton IPT-SIB clinical trial. In this study, we asked if LET_d_ optimization could further improve the TODRs through increased RBEs. We found that the LET-optimized plans did indeed have higher TODRs than SFO or IPT-SIB plans. As a confirmation of previous techniques, we found the IPT-SIB plans to have increased TODRs over SFO plans. Although the IPT-SIB patient outcomes are expected to be higher than from SFO based on physical dose, the increase in LET_d_ only results in RBE increases of 2–3%, and TODR percent difference increases are consistent with RBE = 1.1. LET_d_ optimization, however, achieved LET_d_ in target volumes of ~4 keV/µm, compared to ~2 keV/µm in SFO and IPT-SIB plans, which resulted in RBEs ranging from 1.22 with the McMahon model up to 1.52 with the Du145 model. With the SFO target volumes having a Du145 RBE of about 1.34, LET_d_ optimization results in about an 18% increase, which would be clinically significant. DVHs show, however, that the IPT RBE-dose in the IPT-SIB plan is similar to that in the LET-optimized plan. Because the physical dose is escalated in the IPT-SIB plans compared to the LET-optimized, the nominal RBE-dose to the IPT volume is comparable even though the RBE enhancement in the IPT-SIB plan is not as high. If tumor control is driven by RBE-dose to the IPT, IPT-SIB and LET-optimized plans could have similar 5-year FFBPs. However, because the TODRs increased, the LET-optimized target volume RBE-dose could be escalated even higher without significant increases in OAR toxicities. While LET_d_ optimization resulted in higher LET_d_ in the bladder and rectum V70 regions, which could potentially increase toxicities, there are two factors that may mitigate this: (1) the size of the V70 regions generally decreased by single-digit percentages for the rectum; and (2) with an increase in the TODRs, the dose to the OARs could be reduced even while escalating the dose to the target volumes. The part of the bladder inside the PTV, however, is expected to receive a higher RBE-dose due to the high LET_d_. Considering all of this and assuming a direct correlation between the RBE-dose and patient outcomes, LET-optimized plans are expected to produce a higher 5-year FFBP and lower OAR toxicities than either SFO or IPT-SIB plans. 

### 4.2. Treatment Planning Considerations for LET_d_ Optimization

It is important to note that this study aims to demonstrate the potential for improved patient outcomes through increased LET-dependent RBE, not to provide clinical treatment planning recommendations. There are several factors that still need to be fine-tuned. The largest factor to be addressed is density uncertainty robustness, which is innately a challenge in LET-optimized plans. Unlike SFO plans in which the field is the composite of two opposing beams’ spread-out Bragg peaks (Figure 2 left), for which the field remains flat even with a density perturbation, LET-optimized plans create the field on the distal edges of each beam. Because of the gradient of the distal edges, even small density perturbations can result in large changes in target coverage. This effect is mitigated by creating less steep distal edges, but this also decreases the LET_d_. Hence, the antagonism between high LET_d_, uniform dose, and robustness has recently been dubbed the LET_d_ trilemma [52]. In spite of this, we found that even the LET-optimized plans with “watered down” LET_d_ (~3–3.5 keV/μm) to achieve better robustness still outperform SFO plans in terms of TODRs and minimum average RBE-dose coverage. There is, therefore, no reason to think that the LET_d_ trilemma would hinder the creation of clinically acceptable LET-optimized plans, provided an optimal balance of the three factors is determined. Reducing the density uncertainty, on the other hand, could abate the need to “water down” the LET_d_. Proton radiography [53], by shooting protons through the patient and measuring the residual range, can be used to estimate stopping powers more accurately and could allow for robust optimization using smaller density uncertainties, which would also allow for higher LET_d_. Alternatively, it is possible that the LET-dependent RBE-dose could be robustly optimized instead, in which case uniform physical dose is not a consideration. The challenge of this is creating accurate RBE models, which is described below.

Beam arrangement is another factor that can be optimized. Parallel opposed beams were used throughout this study as SFO and IPT-SIB plans are designed this way. As Figure 10 shows, however, four-beam arrangements can result in increased LET_d_, TODRs, and minimum average RBE-dose while achieving greater density uncertainty robustness. As the anterior oblique beams contribute to decreasing bladder TODR, these two beams could be made parallel opposed again. Alternatively, proton arc therapy could be performed in which the optimizer determines the optimal weighting for each gantry angle. An additional benefit that was not formally evaluated is femoral head sparing. While SFO and IPT-SIB plans had femoral head doses well below the clinical dose goals, the two-beam LET-optimized plans are around or a little above the 50 Gy/55 Gy relative and absolute volume dose goals (see increased entrance dose in Figure 2 left); four-beam treatment completely resolves this issue. One additional factor that still needs to be considered is urethral sparing, as it is in the center of the prostate and receives the increased LET_d_. The LET_d_ in the urethra could be reduced to decrease the RBE, or LET_d_ could be maintained while dropping the physical dose, both of which would slightly decrease the target volume RBE-dose. However, if the urethra α/β is similar to that of the bladder, the McNamara model predicts the RBE would stay lower than for the prostate with its α/β of 1.5 Gy, and potentially, the treatment technique would not need to be adjusted.

### 4.3. RBE Modeling

All RBE models, including RBE = 1.1, predicted increases in the TODRs from SFO to IPT-SIB to LET-optimized plans. Even though the different models predicted a wide range of RBEs, the three LET-dependent RBE models all predicted roughly the same percent differences in TODRs between plans within 2.5%. This demonstrates that although the exact magnitude of biological effectiveness may be difficult to predict, it may be possible to predict how a given plan technique will increase biological effectiveness relative to other plans. RBE = 1.1 well approximated the TODR percent differences between SFO and IPT-SIB plans that were predicted with the LET-dependent RBE models, which could mean that any improvements in patient outcomes may be adequately described by RBE = 1.1. This is consistent with current recommendations for the continued use of RBE = 1.1 [22]. However, for LET-optimized plans, which may have ~2 keV/µm higher LET_d_ than SFO or IPT-SIB plans, RBE = 1.1 greatly underestimated the percent differences in TODRs predicted using the LET-dependent RBE models. Additionally, considering LET-dependent RBE models when evaluating density uncertainty robustness showed that the minimum average RBE-dose stayed above that of SFO plans for all LET_d_ evaluated, while the RBE = 1.1 minimum average RBE-dose dropped unacceptably low in some of the evaluations (Figure 10, see D95 spread). Thus, as treatment planning enters the paradigm of LET_d_ optimization, RBE = 1.1 will not be adequate, and LET-dependent RBE models will be a necessity.

The necessity of LET-dependent RBE models is challenged by the extraordinary amount of biological variability in the data used to fit the models. Generalizability, in principle, should be a goal in RBE modeling. However, the McNamara model (against which the McMahon model was validated) has too much variability in the model fit (refer to McNamara et al. [42]) to be accurately applied in all tissues. The McNamara model was only fit with two data points of Du145 at one value of LET_d_ and underpredicted the empirical RBEs for Du145 in multiple studies [46,47]. The question is, why do we expect models minimally informed by prostate cancer to accurately predict prostate cancer outcomes? It is assumed that all tissues with the same ${\left(\alpha /\beta \right)}_{x}$ will respond the same to LET_d_, although this is not necessarily true. In diagnostic imaging artificial intelligence, models are developed for modality-specific individual disease sites [54,55,56]. We believe that RBE models should also be developed using disease-specific data. The Du145 model in this study resulted in higher RBEs and TODRs than either the McMahon or McNamara models, demonstrating the importance of disease-specific models in treatment planning as this will inform the prescription dose. While a step in the right direction, in vitro cell survival data may still not be representative of patient populations in which tumors are in situ, heterogeneous, and may have varying sensitivities to LET_d_. Ultimately, patient outcomes data from prostate cancer patients treated with PBS proton therapy with varying levels of LET_d_ is needed to develop clinical endpoint-specific RBE models for clinical use.

## 5. Conclusions

LET_d_ optimization resulted in the abutment of the distal edges from opposed beams to yield a LET_d_ of ~4 keV/µm in target volumes and shot through the rectum to keep LET_d_ low. While IPT-SIB plans had a 2–3% increase in RBE over SFO plans, a prostate-cancer-specific LET-dependent RBE model predicted an 18% increase in RBE for LET-optimized plans over SFO plans with RBEs > 1.5. LVHs/DVHs showed large increases in LET_d_ and RBE-dose, respectively, in target volumes, while only modest changes were observed in the OARs. All RBE models, including RBE = 1.1, predicted an increase in TODRs from SFO to IPT-SIB to LET-optimized plans. A robust evaluation of density uncertainty for an example patient showed that decreasing LET_d_ to ~3–3.5 keV/μm may be necessary to achieve acceptable robustness when evaluating dose using RBE = 1.1. Although the RBE = 1.1 dose showed sensitivity to robust perturbations, TODRs and the minimum average RBE-dose (i.e., the average RBE-dose in the +2% density perturbation scenario) remained greater than those of the SFO plan. While RBE = 1.1 may approximate the percent difference in TODRs predicted using LET-dependent RBE models between SFO and IPT-SIB plans, LET-dependent RBE models are a necessity when considering LET_d_ optimization.

## Figures and Tables

**Table 1:** Mean LET_d_ and mean RBE with 95% CIs calculated (for the McNamara model, prescribed fraction (fx) doses were assumed for target volumes and 1.9 Gy(RBE)/fx for V70 regions (average between 70 Gy(RBE) and 78 Gy(RBE) in 39 fractions)). Bladder and rectum V70 RBEs were not calculated with the Du145 model since it is prostate-cancer-specific.

	Mean LET_d_ (keV/µm)	McMahon RBE	McNamara RBE	Du145 RBE
**SFO Plan** PTV PSV IPT (2 Gy(RBE = 1.1)/fx) Prostate Bladder V70 Rectum V70	2.190 [2.153, 2.227] 2.103 [2.067, 2.140] 2.120 [2.088, 2.154] 2.349 [2.281, 2.409] 2.196 [2.136, 2.258]	1.120 [1.118, 1.123] 1.116 [1.114, 1.118] 1.117 [1.115, 1.119] 1.129 [1.126, 1.133] 1.121 [1.118, 1.124]	1.195 [1.193, 1.197] 1.190 [1.188, 1.192] 1.191 [1.189, 1.193] 1.123 [1.121, 1.125] 1.112 [1.110, 1.114]	1.344 [1.341, 1.347] 1.337 [1.334, 1.340] 1.338 [1.335, 1.341] - -
**IPT-SIB Plan** PTV PSV IPT (2.2 Gy(RBE = 1.1)/fx) Prostate Bladder V70 Rectum V70	2.411 [2.324, 2.494] 2.428 [2.352, 2.500] 2.353 [2.280, 2.429] 2.466 [2.351, 2.569] 2.370 [2.233, 2.500]	1.133 [1.128, 1.137] 1.134 [1.129, 1.138] 1.129 [1.125, 1.134] 1.136 [1.129, 1.141] 1.130 [1.123, 1.138]	1.206 [1.202, 1.211] 1.199 [1.196, 1.203] 1.203 [1.199, 1.207] 1.127 [1.123, 1.130] 1.118 [1.113, 1.122]	1.363 [1.355, 1.370] 1.364 [1.358, 1.370] 1.358 [1.352, 1.364] - -
**LET-Optimized Plan** PTV PSV IPT (2 Gy(RBE = 1.1)/fx) Prostate Bladder V70 Rectum V70	3.975 [3.945, 4.004] 4.277 [4.226, 4.327] 4.253 [4.215, 4.286] 3.631 [3.588, 3.673] 2.905 [2.847, 2.967]	1.219 [1.217, 1.220] 1.235 [1.233, 1.238] 1.234 [1.232, 1.236] 1.200 [1.197, 1.202] 1.160 [1.157, 1.163]	1.287 [1.286, 1.289] 1.302 [1.300, 1.305] 1.301 [1.299, 1.303] 1.167 [1.165, 1.168] 1.135 [1.133, 1.137]	1.494 [1.491, 1.496] 1.519 [1.515, 1.524] 1.517 [1.514, 1.520] - -

## Data Availability

Data supporting this study are included within the article. The derived data supporting the findings of this study are available upon reasonable request from the corresponding author, Mark Artz.
